# Cerebral toxoplasmosis associated with the use of immunosuppressive treatments

**DOI:** 10.1590/0037-8682-0720-2021

**Published:** 2022-04-08

**Authors:** Ivone Melo Valadão, Ana Sofia Sequeira, Vanessa Barcelos

**Affiliations:** 1 Hospital Santo Espírito da Ilha Terceira, Internal Medicine Department, Angra do Heroísmo, Azores, Portugal.

A 75-year-old male was admitted to the emergency department with right-sided weakness for 3 weeks and fever (38 °C). His medical history included rheumatoid arthritis treated with adalimumab and methotrexate. Neurological examination revealed right central facial palsy and right brachial hemiparesis with a motor strength of 3/5 in the right extremities. Laboratory tests did not reveal alterations. Serological tests showed positivity for toxoplasma immunoglobulin G (>300 IU/mL) with immunoglobulin M negativity. Brain magnetic resonance imaging (MRI) revealed a centered lesion without significant postcontrast enhancement (Figure 1). Even though the radiological findings were not fully conclusive, they pointed to an infectious cause. A lumbar puncture was performed and the polymerase chain reaction (PCR) for *Toxoplasma gondii* in the cerebrospinal fluid was positive. Despite the atypical imaging appearance, the analysis of the entire clinical picture with PCR for *T. gondii* positivity suggested the diagnosis of cerebral toxoplasmosis. The patient started therapy with pyrimethamine, sulfadiazine, and folinic acid. At discharge, he recovered completely from his neurological deficits. After finishing the treatment, MRI showed improvement in edema with a practically calcified lesion (Figure 2).

**FIGURE 1:**
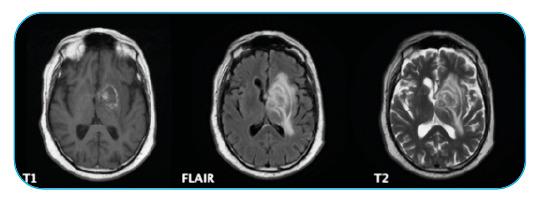
Intraparenchymal lesion involving the striatocapsular and left temporal regions, without significant postcontrast enhancement and showing foci of spontaneous hypersignals in T1. Injury conditions cause molding of the ventricular system and deviation of the midline structures to the left.

**FIGURE 2:**
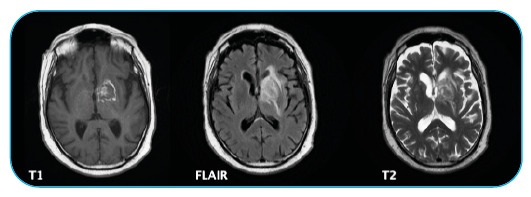
The lesion partially calcified without significant postcontrast enhancement in the left striatocapsular region. The reduction of the edema component, which does not currently extend to the temporal lobe, is more evident in T2 and FLAIR. Reduction of the mass effect on the ventricular system, with the incipient deviation of the pellucid septum to the right persisting.

The clinical signs of cerebral toxoplasmosis are vague and may mimic many other conditions^1^. Therefore, imaging plays an essential role in diagnosis^2^. In 70-80% cases, the lesions are enhanced in a homogeneous or ring pattern with contrast^3^. 

This clinical case intends to alert the scientific community to a difficult-to-diagnose condition that presents various atypical radiological findings.

## References

[1] Elsheikha HM, Marra CM, Zhu XQ (2020). Epidemiology, Pathophysiology, Diagnosis, and Management of Cerebral Toxoplasmosis. Clin Microbiol Rev.

[2] Pulivarthi S, Reshi RA, McGary CT, Gurram MK (2015). Cerebral toxoplasmosis in a patient on methotrexate and infliximab for rheumatoid arthritis. Intern Med.

[3] Robert-Gangneux F, Sterkers Y, Yera H, Accoceberry I, Menotti J, Cassaing S (2015). Molecular diagnosis of toxoplasmosis in immunocompromised patients: a 3-year multicenter retrospective study. J Clin Microbiol.

